# Results of extracorporeal life support implementation in routine clinical practice: single center experience

**DOI:** 10.3325/cmj.2014.55.600

**Published:** 2014-12

**Authors:** Bojan Biočina, Mate Petričević, Dražen Belina, Hrvoje Gašparović, Lucija Svetina, Sanja Konosić, Alexandra White, Višnja Ivančan, Tomislav Kopjar, Davor Miličić

**Affiliations:** 1 Department of Cardiac Surgery, University Hospital Center Zagreb, University of Zagreb School of Medicine, Zagreb, Croatia; 2 Department of Anesthesiology, University Hospital Center Zagreb, Zagreb, Croatia; 3 Department of Cardiovascular Diseases, University of Zagreb School of Medicine, University Hospital Center Zagreb, Zagreb, Croatia

## Abstract

**Aim:**

To describe our experience in the clinical application of extracorporeal life support (ECLS) and analyze whether ECLS leads to acceptable clinical outcomes in patients with cardiac failure.

**Methods:**

Data from clinical database of University Hospital Center Zagreb, Croatia, on 75 patients undergoing ECLS support from 2009 to 2014 due to cardiac failure were retrospectively analyzed. Outcomes were defined as procedural and clinical outcomes. ECLS as a primary procedure and ECLS as a postcardiotomy procedure due to inability to wean from cardiopulmonary bypass were analyzed.

**Results:**

ECLS was used in 75 adult patients, and in 24 (32%) of those procedural success was noted. ECLS was implemented as a primary procedure in 36 patients and as a postcardiotomy procedure in 39 patients. Nine out of 39 (23.08%) patients had postcardiotomy ECLS after heart transplantation. Bleeding complications occurred in 30 (40%) patients, both in primary (11/36 patients) and postcardiotomy group (19/39 patients). ECLS was established by peripheral approach in 46 patients and by central cannulation in 27 patients. In 2 patients, combined cannulation was performed, with an inflow cannula placed into the right atrium and an outflow cannula placed into the femoral artery. Eleven patients treated with peripheral approach had ischemic complications.

**Conclusion:**

ECLS is a useful tool in the treatment of patients with refractory cardiac failure and its results are encouraging in patients who otherwise have an unfavorable prognosis.

The idea of extracorporeal life support (ECLS) became a reality with the introduction of the heart-lung machine by Gibbon in 1954 (1). However, only in 1971 the first successful extracorporeal membrane oxygenation treatment was described (2). Since then continuous technological improvements have led to the growing interest in this type of treatment (3).

ECLS may significantly improve the level of patient care in patients with acute and chronic heart failure. It is an advanced form of treatment and the patient management is carried out by a multidisciplinary team of cardiac surgeons, anesthesiologists, and cardiologists.

There are two main groups of indications for ECLS: those not related and those related to cardiac surgery. Primary ECLS procedures are not related to cardiac surgery but to acute cardiorespiratory failure arising from underlying cardiac disease. Postcardiotomy ECLS procedures may be considered as a secondary procedure and are directly related to cardiac surgery procedures. These procedures are indicated after cardiac surgery procedures when patients cannot be weaned from cardiopulmonary bypass or when there is low cardiac output syndrome in the early postoperative phase. Postcardiotomy cardiogenic shock occurs in up to 6% of cardiac operations (4-6), with only 25% of those patients surviving to hospital discharge (4). Therefore, postcardiotomy cardiogenic shock is one of the most difficult and resource-consuming conditions, as it is associated with particularly high mortality rates. Since the risk profiles of patients scheduled for cardiac surgery procedures are continuously worsening, one may expect an increase in the number of patients requiring ECLS peri-operatively. Although these patients have only 25% of survival to hospital discharge (4), one should be aware that they would have nearly 100% mortality if they were not placed on ECLS. Although early ECLS results for postcardiotomy cardiogenic shock were found to be poor (5,7), they have been improved by continuous technological advances (5,8-10). Primary ECLS procedures may also play a significant role in cardiac surgery patients. In general, ECLS restores body perfusion and allows different treatment modalities. This may help optimize a patient’s clinical condition while waiting for a heart transplant. If a patient is not considered a suitable candidate for a heart transplant, ECLS may provide a bridge to long-term mechanical circulatory support. However, if ECLS is intended to bridge a patient to recovery (for instance in patients with acute myocarditis), then ECLS support may be considered as a treatment per se.

ECLS may be used as either; 1) bridge to recovery, 2) bridge to transplant, 3) bridge to decision, or 4) bridge to intermediate or long term support. “Bridging concept” is important because a lack of exit strategy (eg, reason for bridging) may actually suggest a possible contraindication for ECLS.

ECLS may play a significant role in patients with acute cardiogenic shock prior to scheduled cardiac surgery procedures such as intermediate to long term mechanical circulatory support, heart transplant, or other cardiosurgical procedures depending on the preexisting condition.

Preoperative patient optimization using ECLS improves the outcomes of the level 1 Interagency Registry for Mechanically Assisted Circulatory Support (INTERMACS) patients receiving a permanent ventricular assist device (11). This “bridge-to-bridge” concept consisted of preoperative stabilization of level 1 INTERMACS patients using ECLS a few days prior to ventricular assist device implantation. ECLS significantly improved renal, hepatic, and pulmonary functions (11).

In addition to this, preoperative ECLS support may significantly improve outcomes in patients presenting with acute myocardial infarction (AMI) complicated with ventricular septal defect (VSD) and cardiogenic shock (12). Preoperative ECLS provided hemodynamic support, ensured end-organ perfusion, helped the myocardium to recover and allow scar tissue to be formed by reducing sheer forces, thus leading to more favorable outcomes (12).

For both primary and postcardiotomy indications, our team generally prefers combined support to the heart and lungs (ECLS) rather than isolated mechanical circulatory support to the failing heart. ECLS can be implanted bedside, providing support to both the heart and the lungs simultaneously. Nowadays ECLS is available in many centers that do not perform ventricular assist device implantation. In such centers, patients should be preoperatively stabilized and transferred on support to tertiary care centers for further treatment. To facilitate this process, it would be necessary to establish an ECLS network with ECLS centers cooperating with tertiary centers that perform long term mechanical circulatory support, heart transplant, as well as complex surgical corrections. In this article, we describe our experience in the clinical application of extracorporeal life support (ECLS) and aim to analyze whether ECLS leads to acceptable clinical outcomes in patients with cardiac failure.

## Methods

ECLS was introduced at the Department of Cardiac Surgery, University Hospital Center Zagreb in 1987. From 1987 until 1995 only 12 patients were supported with MCS (ECLS 6 patients, LVAD 6 patients) but only 3 survived (1 patient on ECLS and 2 patients on LVAD). The mechanical circulatory support (MCS) program was established in 2008. Upon commencement of this program, a patient database was constructed, with anonymized and coded patients’ records.

This study presents retrospective analysis of data collected from 2009 to 2014, and it was approved by the University of Zagreb School of Medicine Ethics Committee.

Typical clinical scenarios for ECLS initiation were as follows: postcardiotomy cardiac failure, acute exacerbation of chronic severe heart failure, acute heart failure due to myocardial infarction, and cardiac arrest requiring cardiopulmonary resuscitation (CPR). Whenever possible, ECLS management was in line with the guidelines provided by ECLS working group (10).

Patients were classified according to INTERMACS registry (13). We defined outcomes as procedural and clinical outcomes. A procedural outcome was considered as success if ECLS successfully bridged the patient to recovery, long-term mechanical circulatory support, or heart transplant. Therefore, procedural success was defined as adequate employment of intentional strategy for ECLS. A procedural outcome was considered as failure if ECLS did not achieve the previously defined target. Procedural success may ultimately result in adverse outcomes such as death, but procedural failure is inevitably associated with adverse outcomes. Clinical outcomes were defined as the following conditions after 30 days follow up: alive, alive on support, and dead.

### Implantation technique

ECLS was established by either central or peripheral approach depending on the underlying pathology. For primary ECLS procedures, a femoral route was preferred. The first modality for cannula insertion was Seldinger technique. In case of failed attempts of Seldinger technique, surgical exploration of the femoral vessels was performed. Postcardiotomy ECLS was established via a median sternotomy. Central cannulation sites were the right atrium for inflow cannula and the ascending aorta for outflow cannula.

### Components of the ECLS circuit

**Figure 1:**
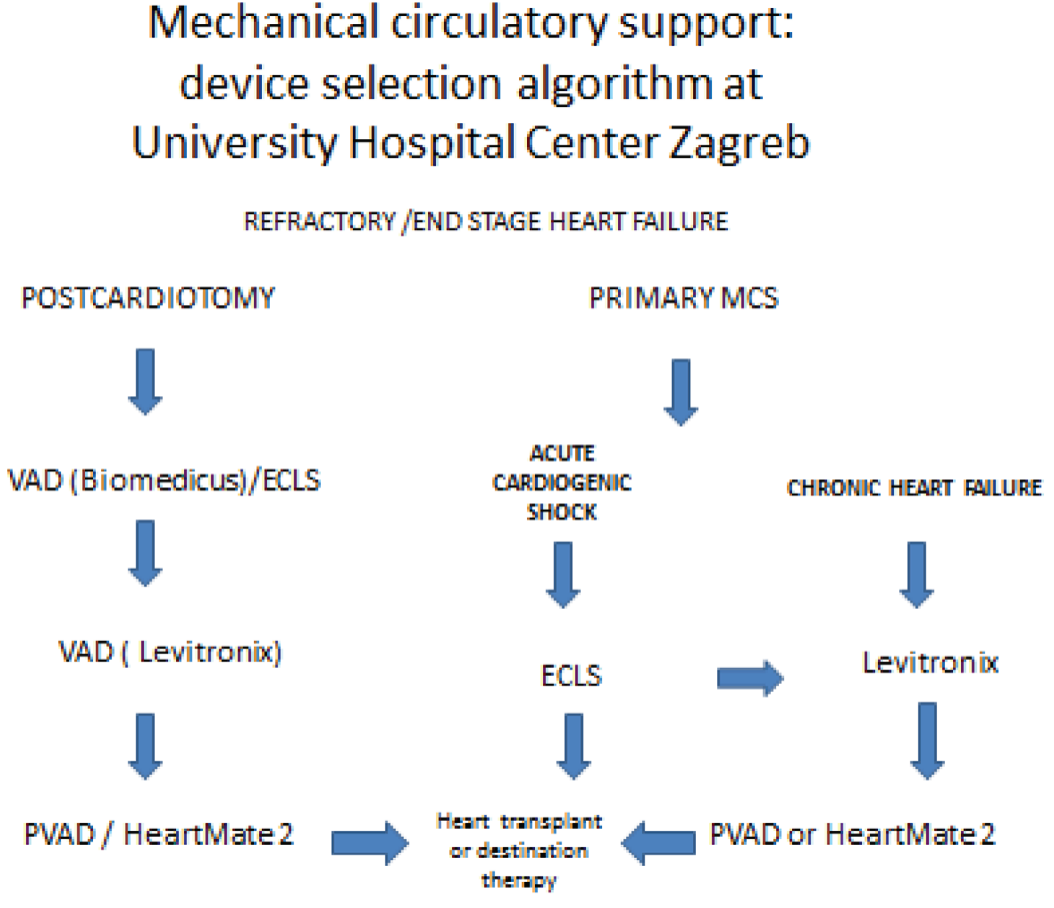
Mechanical circulatory support at the Department of Cardiac Surgery, University Hospital Center Zagreb. Device selection algorithm. MCS – mechanical circulatory support; VAD – ventricular assist device; ECLS – extracorporeal life support; PVAD – paracorporeal ventricular assist device.

The organization of ECLS was an integral part of the institutional algorithm on the device use and the type of support selection (Figure 1). Several components of the ECLS circuit are mandatory to initiate ECLS support. In brief, the ECLS circuit consists of an inflow and outflow cannulas, blood pumping device, and oxygenator. The following centrifugal pumps were used for blood pumping: Maquet Rotaflow centrifugal blood pump (Maquet Cardiovascular LCC, San Jose, CA, USA), Medtronic Bio-Medicus centrifugal pump (Medtronic Inc, Minneapolis, MN, USA), and Levitronix Centrimag (Levitronix, Boston, MA, USA). Applied hollow fiber oxygenators were Maquet Quadrox and Euroset, as well as Medtronic IE 4500 flat sheet type oxygenator in a few cases. Biocompatible surface coating lines were used.

### Anticoagulation protocol

All patients in a non-operative setting (primary procedures) received a bolus dose of 50-100 IU/kg unfractionated heparin (UFH) before cannulation, and the dose was modified based on patients’ pre-ECLS coagulation status and tendency to bleed. When activated clotting time (ACT) dropped below 300 s, a continuous UFH infusion was initiated at a rate of 10-20 IU/kg/h. In the postcardiotomy setting, a bolus dose was required only if heparin was already fully reversed by protamine. Furthermore, a continuous UFH infusion at a rate of 10 UI/kg/h was usually initiated after 24 hours (and/or when the chest tube drainage was less than 4 mL/kg/h). The UFH infusion was then adjusted based on the results of anticoagulation monitoring. These included activated partial thromboplastin time (aPTT – measured every 8 hours, with the target value 1.5-2.5 × normal), ACT (measured every 4 hours, with a target value of 180-200 s), and anti Xa level (target value 0.4-0.7 IU/mL). Standard laboratory tests were obtained twice daily and their values were maintained at near normal values (hematocrit >0.30, platelets >100 × 10^9^/L, fibrinogen >2 g/L, international normalized ratio (INR) 0.8-1.1, antithrombin III (ATIII)>60%). However, since most thomboembolic and hemorrhagic events occur despite standard laboratory tests being within the reference ranges (14), evaluation of viscoelastic properties of blood clots was performed using rotational thromboelastometry on a daily basis (14). In patients with clinical suspicion to heparin-induced thrombocytopenia, fondaparinux (0.07-0.1 mg/kg daily, with anti Xa level target peak value of 1.0-1.2 IU/mL) was used instead of UFH.

### Weaning protocol

Usually, the first evidence of myocardial recovery is increased pulsatility of an arterial waveform. If this is accompanied by an improvement in echocardiographic appearance of cardiac function and the absence of compromised pulmonary blood oxygenation, the patient is considered eligible for an ECLS weaning trial (15). Before the weaning trial was undertaken, inotropes/vasopressors of choice (dobutamine, epinephrine, milrinone, levosimendan, norepinephrine) were administered in moderate doses, volume status was optimized (best guided by echocardiography), and ACT was targeted to at least 220 s (16). While ECLS flow was decreased in increments by 20% every few hours, echocardiographic, hemodynamic, and laboratory parameters were constantly evaluated. Flow was reduced in a stepwise manner to 1-1.5 L/min. If the patients were not demonstrating any signs of hemodynamic compromise (stabile arterial blood pressure, adequate cardiac output, mixed venous saturation (O2)>60%, central venous pressure <12 mm Hg, acceptable acid base status, lactate <3), they were usually weaned form ECLS and the cannulas were removed. However, if they did not tolerate the proposed decrease in mechanical support, the flow was restored to a higher value. Occasionally weaning off mechanical support was not possible, and bridging to ventricular assist device or heart transplant was considered.

## Results

**Figure 2:**
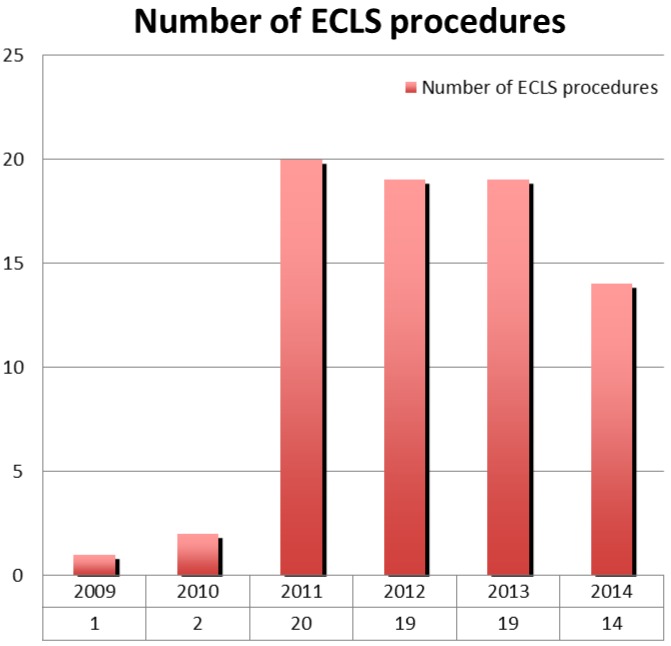
The number of extracorporeal life support (ECLS) procedures performed per year.

From 2009 to 2014, 133 adult patients underwent mechanical circulatory support with 165 mechanical circulatory support procedures performed (Figure 2). Several patients underwent multiple procedures. ECLS was used most frequently – in 75 adult patients, 53 of whom (70.67%) were INTERMACS level 1 patients and 22 (29.33%) were INTERMACS level 2 patients (Table 1).

**Table 1:** Baseline demographics, clinical characteristics, and surgery data*^†^

Demographic data	All patients (n = 75)
Age (years)	58 (49-65)
Men	47 (63)
Women	28 (37)
Body weight (kg)	78 (72-87)
Body height (cm)	171 (166-178)
BMI (kg/m^2^)	26 (24-30)
BSA (m^2^)	1.9 (1.8-2.1)
Clinical characteristics	
Primary ECMO	36 (48)
Postcardiotomy ECMO	39 (52)
INTERMACS 1	52 (69)
INTERMACS 2	23 (31)
INTERMACS 3 and above	0 (0)
Surgery data	
LA venting to ECMO “inflow”	4 (5)
Re-exploration for bleeding	30 (40)
Implantation between 2009 and 2011	23 (31)
Implantation between 2012 and 2014	52 (69)

*Abbreviations: BMI – body mass index; BSA – body surface area; ECMO – Extracorporeal membrane oxygenation; INTERMACS - Interagency Registry for Mechanically Assisted Circulatory Support; LA – left atrium.

†Data are presented as medians and interquartile ranges. Categorical variables are shown as absolute numbers with percentages.

Single ECLS procedures were performed in 59 (78.7%) patients. ECLS procedures that were part of a multi-procedural MCS treatment were performed in 16 patients (21.3%). All 16 patients were upgraded to longer-term mechanical circulatory support procedures. Of those, 7 patients underwent left ventricular assist device implantation (3 Heart Mate II and 4 Levitronix Centrimag), 6 were placed on right ventricular assist device, and 3 were placed onto biventricular support (1 patient on Thoratec pVAD and 2 patients on Levitronix Centrimag).

ECLS was implemented as a primary procedure in 36 patients and as a postcardiotomy procedure in 39 patients. Nine out of 39 (23.08%) patients had postcardiotomy ECLS after heart transplantation. The rest of postcardiotomy procedures were performed after heart valve surgery – one or more valves (15 patients), coronary artery surgery (7 patients), surgery for aortic dissection (1 patient), surgery for postinfarction ventricular septal defect (4 patients), Heart Mate 2 implantation (1 patient), and Bentall procedure (2 patients). It is very important to notice that 11 heart transplant patients were treated with ECLS as a primary procedure. Of these, 6 were bridged with ECLS to heart transplant. In summary, 20 patients from the heart transplant program were treated with ECLS support (Table 2).

**Table 2:** Procedural and clinical outcomes. Patients were divided according to type of procedure

Outcome, n (%)	Primary procedure n = 36	Postcardiotomy procedure n = 39	Total procedures n = 75
Procedural:			
success	12 (33)	12 (31)	24 (32)
failure	24 (67)	27 (69)	51 (68)
Clinical:			
alive	8 (22)	11 (28)	19 (25)
alive on support	4 (11)	1 (3)	5 (7)
dead	24 (67)	27 (69)	51 (68)

Bleeding events occurred in 30 (40%) patients, both in primary (11/36 patients) and postcardiotomy group (19/39 patients). Characteristics of bleeding events varied between patients. In general, such events occurred in the form of excessive mediastinal bleeding requiring surgical re-exploration, bleeding related to cannulation site in femoral arteries, retroperitoneal bleeding due to cannula misplacement, gastrointestinal bleeding, and bleeding from the nasopharynx requiring surgical tamponade. Cardiac decompression due to left ventricular distention while on ECLS support was performed in 4 out of 75 patients (5.3%).

Peripheral cannulation was performed in 46 and central cannulation in 27 patients. Combined approach was performed in 2 patients, with an inflow cannula placed into the right atrium and an outflow cannula placed into the femoral artery. In patients with peripheral approach, ischemic complications occurred in 11 cases. Three out of 11 patients with vascular complications developed gangrene with diffuse lower limb ischemia.

## Discussion

ECLS is a useful tool in the treatment of patients with refractory cardiac failure. Patients on ECLS (17) show improved outcomes, as well as favorable long-term outcomes and good quality of life of survivors (17). However, ECLS is related to a considerable rate of complications that require comprehensive and multidisciplinary management.

We used ECLS in 75 adult patients and procedural success was noted in 32%. Bleeding was the major complication, which occurred in 40% patients.

Despite appropriate ECLS, a certain number of patients develop left ventricular edema (18). We found that cardiac decompression due to left ventricular distention while on ECLS was performed in 4 out of 75 patients (5.3%). In these patients, a left atrial vent was placed through the right upper pulmonary vein and connected via a “Y” connector to the tubes connected to the inflow cannula. This measure requires a sternotomy, but there is evidence that minimally invasive approach for cardiac decompression is feasible. Percutaneous transseptal left atrial decompression (19) and subxiphoid surgical decompression of the left ventricle (20) have already been described. A solution to avoid high-risk septostomy or surgical venting may be a percutaneously inserted ventricular assist device (Impella; Abiomed, Aachen, Germany) (21). In addition to this, general measures should be considered to prevent left ventricular distension on ECLS, such as afterload reduction, the use of inotropes, eg, epinephrine, calcium sensitizer levosimendan, arterial vasodilators, avoidance of volume overload, and hemofiltration. Aortic valve insufficiency and inadequate venous drainage may precipitate ventricular distention on ECLS (18).

Even though ECLS has been successfully used for well over the 30 years, the anticoagulation management in these patients remains controversial. The contact between blood and nonbiologic surface of an extracorporeal circuit provokes a massive acute response leading to the activation of both procoagulant and anticoagulant components and their subsequent consumption. The use of a systemic anticoagulant drug is mandatory in order to prevent the development of thromboembolic events, however it is challenging to balance its effect against the risk of bleeding (22). The preferred anticoagulation agent in the majority of ECLS centers is UFH, while direct thrombin inhibitors are seldom primarily used (16).

In this study, bleeding events occurred in 30 (40%) patients. Bleeding events are common in patients on ECLS support and may stem from the cannula insertion sites, especially the artery. Additionally, bleeding may occur spontaneously (intracranial and gastrointestinal bleeding) or may be related to minor interventions (23). Even diffuse intraoperative bleeding coupled with massive transfusion requirements leading to delayed chest closure may be considered as a bleeding event. Excessive bleeding and massive transfusions may affect up to 80% of patients on ECLS support (24). Since these patients are thrombocytopenic and have consumption coagulopathy, we strongly suggest point-of-care monitoring of hemostasis using thromboelastometry and platelet function testing (25). In our center, we perform thromboelastometric measurements on a daily basis for the first seven days following ECLS procedure. Prior to surgical intervention, it is of utmost importance to search for any hemostatic disorders and treat them selectively using rotational thromboelastometry (25).

ECLS support has a very important role in cardiac surgery patients who are unable to wean from cardiopulmonary bypass (CPB). The prevalence of postcardiotomy cardiogenic shock ranges from 0.2 to 6% (4). Failure to wean from CPB remains the most common indication for ECLS in cardiac failure. It is noteworthy that for postcardiotomy cardiogenic shock, the survival rate was only 25% even with the use of a ventricular assist devices (26,27). Since postcardiotomy cardiogenic shock may mandate combined heart and lung support, we advise the use of ECLS, although there is scarce evidence on the usefulness of ECLS for postcardiotomy cardiogenic shock treatment and the studies are limited by a small number of patients and retrospective data collection. Nevertheless, survival rates ranging from 16%-76% suggest that it might be a useful tool in this type of treatment (5,6,28-32). These results may be further improved by timely application (4). Our survival rate was 30.76%. In addition, clinical decision making should be guided by a model that predicts poor outcomes after postcardiotomy. However, to develop such a model, registries of data pooled from all centers should be established. Such approach could compensate for the lack of publications reporting poor outcomes and lead to meaningful conclusions based on “real-world” results.

In our study, 9 out of 39 (23.08%) patients received postcardiotomy ECLS after heart transplantation. This proportion is similar to 19% reported by ELSO registry. In addition, 11 patients with heart transplants received primary ECLS. In general, 20 out of 75 ECLS patients (26.67%) underwent heart transplantation, which shows successful integration of mechanical circulatory support and heart transplant. According to Santise et al (33), 15.7% of heart transplant patients develop primary graft failure requiring ECLS support, with weaning and discharge rates of 84% and 53%, respectively, and similar results were reported by Lehmann et al (34) and Marasco et al (35). Primary ECLS enhanced the pool of potential heart recipients by increasing the number of patients at high risk. On the other hand, postcardiotomy ECLS was an acceptable last resort option for the otherwise hopeless patients with fatal graft failure after heart transplantation (35).

In patients requiring ECLS support, approach to cannulation should be personalized and based upon clinical condition. For postcardiotomy procedures, intrathoracic cannulation of the right atrium and ascending aorta seems to be the most appropriate approach. In such cases, one should be aware of the possibility of excessive postoperative bleeding, which may sometimes mandate delayed chest closure with subsequent re-explorations. For primary ECLS procedures, the preferable procedure is peripheral cannulation. However, such an approach is associated with arterial vascular complications including ischemic and thrombotic complications. In addition, there is the problem of bleeding complications. Peripheral cannulation may be performed by percutaneous Seldinger’s technique or an open surgical approach. Whenever feasible, we prefer Seldinger’s technique under ultrasound/Doppler guidance. Ultrasound may additionally help in choosing the cannula for arterial cannulation, so that cannula size can be adapted to the vessel size. If percutaneous femoral cannulation proves to be difficult to perform, we regularly apply the open surgical approach, and if the femoral artery is noted to be small, 8 cm Dacron graft is connected to the femoral artery via termino-lateral anastomosis.

Among patients with peripheral arterial cannulation, we observed ischemic complications in 11 cases, 3 out of whom developed gangrene with diffuse lower limb ischemia. Therefore, it is extremely important to frequently verify the pulsatility of the anterior and posterior tibial artery and to restore the limb perfusion promptly after signs of hypoperfusion have been observed. Our rate of vascular complications is comparable to the rates in the literature, which range between 3.2% and 28% in femoral cannulation sites (36).

Aside from ischemic complications, one should be aware of hyperperfusion syndrome presented as an edematous limb that is hyperemic and warm to the touch. Hyperperfusion syndrome occurs in the limb ipsilateral to the side of arterial cannulation. Hyperperfusion syndrome has been more frequently associated with ECLS support, with axillary artery side graft technique in up to 24.7% of patients (37). This syndrome may cause compartment syndrome, leading to secondary ischemic complications that may require prophylactic fasciotomy or even limb amputation. Fortunately, none of our patients had hyperperfusion syndrome.

Although associated with considerable complication rates, ECLS is a powerful tool for treatment of patients with refractory cardiorespiratory failure. To achieve better results and establish ECLS as an integral part of cardiorespiratory failure treatment algorithm, multidisciplinary management and extensive clinical experience are required, together with careful patient selection and pooling of evidence.
